# Effects of pre-oxidation on the corrosion behavior of pure Ti under coexistence of solid NaCl deposit and humid oxygen at 600 °C: the diffusion of chlorine

**DOI:** 10.1038/s41598-020-73034-y

**Published:** 2020-10-01

**Authors:** Lei Fan, Li Liu, Yuhai Lv, Hao Wang, Anqing Fu, Juntao Yuan, Ying Li, Fuhui Wang, Chengxian Yin

**Affiliations:** 1 State Key Laboratory for Performance and Structure Safety of Petroleum Tubular Goods and Equipment Materials, CNPC Tubular Goods Research Institute, NO. 89, Jinyeer Rode, Xi’an, 710077 Shaanxi China; 2 Key Laboratory for Anisotropy and Texture of Materials (MoE), School of Materials Science and Engineering, Northeastern University, NO. 3-11, Wenhua Road, Shenyang, 110819 China; 3 Institute of Metal Research, Chinese Academy of Sciences, NO. 62, Wencui Road, Shenyang, 110016 Liaoning China; 4 NO.1 Gas Production Plant of Changqing Oilfield Company, Jingbian, 718500 Shaanxi China

**Keywords:** Metals and alloys, Coarse-grained models

## Abstract

The effect of pre-oxidation on the corrosion behavior of pure Ti covered with a solid NaCl deposit in the humid O_2_ flow at 600 °C is studied. The oxide scale, formed by pre-oxidation, protects the substrate from the NaCl induced corrosion during the initial stage. However, the corrosion of the pre-oxidized sample is severely accelerated by solid NaCl after an incubation period. The chlorine, generated from the decomposition of solid NaCl, diffuses into the oxide/substrate interface as ions during the incubation period, which was observed by ToF–SIMS. The chlorine at the oxide/substrate interface induces the fast corrosion after the incubation period although the pre-oxidation scale is complete and compact.

## Introduction

Compressor blades of airplanes and ships suffer severe corrosion in marine environment, which is mainly due to the fact that marine air contains abundant salts (especially NaCl) and water vapor. At the temperature of 300–600 °C where compressor blades operate, NaCl appears in solid state and gets deposited on the metal surface. The corrosion of several metals and alloys of compressor blades gets accelerated under a synergistic effect of the solid NaCl deposit and humid oxygen.

Extensive works have been done on the corrosion behavior of several metals and alloys of compressor blades. The authors in paper^1–11^ demonstrated that the corrosion behavior of pure Fe, pure Cr, Fe–Cr alloy, Ti alloy and Ni alloy at 600 ˚C under a deposit of solid NaCl deteriorated sharply. Similarly, the authors investigated the influence of KCl on the oxidation of the 304-type (Fe18Cr10Ni) austenitic stainless steel at 600 ˚C in 5% O_2_ and in 5% O_2_ + 40% H_2_O in paper^12–17^, and the results showed that small additions of potassium chloride strongly accelerated high temperature corrosion. The fast corrosion is mainly due to a chemical reaction of the solid NaCl/KCl deposit with oxides. This reaction destroys the protective oxide scale on the surface and forms a porous non-protective scale (i.e., the scale has numerous holes and voids^1,6,8,9,15,17^). It is also reported in these investigations that the key factor for the fast corrosion is the chlorine, HCl or Cl_2_, which cyclically reacts with the substrate at the oxide/substrate interface. The gaseous molecule of chlorine, which is formed during the chemical reaction of solid NaCl and oxide^1,8,18^, diffuses inward quickly through macro defects (like cracks and holes) in the scale of corrosion products. The chlorine then reacts cyclically with the substrate to generate volatile products (for example CrCl_3_) which break the protective scale ^1,3,6,8^.

In our previous work^11^, we observe that the scale of corrosion products of Ti60 alloys can be divided into an outer the corrosion layer and an inner corrosion layer after exposure at 600 ˚C in the environment of NaCl + H_2_O + O_2_. The chlorine exists in a Ti-Cl bond which is formed by replacing O with Cl within lattices of Ti oxides of the inner corrosion layer. We think the chlorine can diffuse inward as ion. In this case, Cl^-^ comes from the decomposition of solid NaCl and the residual sodium reacts with oxides on the surface to form metallic acid salts (like Na_2_CrO_4_^1^ and Na_4_Ti_5_O_12_^10^). In order to make clear the corrosion mechanism, we need to first figure out the state of the chlorine and study its diffusion in the scale of corrosion products, especially during the destruction process of the scale of protective oxide, like TiO_2_ and Cr_2_O_3_.

When pure Ti is oxidized in the pure O_2_ condition at 600 ˚C, a compact and even oxide scale forms on the surface^10^. This oxide scale can simulate the passive film to protect the substance from the corrosive environment. When the pre-oxidized pure Ti is exposed in the NaCl + H_2_O + O_2_ environment, this oxide scale can slow down the destruction process of the protective passive film such that the behavior of the solid NaCl can be investigated in detail. Thus, to clarify the micro-mechanism under the specific environment of NaCl + H_2_O + O_2_, we need to study the effects of pre-oxidation on the corrosion behavior of pure Ti.

In this paper, we investigate the effects of pre-oxidation on the corrosion behavior of pure Ti underneath a solid NaCl deposit in humid O_2_ flow at 600 ˚C, using scanning electron microscope (SEM) equipped with an energy dispersive spectrometer (EDS), X-ray diffraction (XRD) and time of flight-secondary ion mass spectrometry (ToF–SIMS). After examining the detailed properties of the corrosion products and the diffusion of chlorine in the oxide scale, we discuss possible micro acceleration mechanisms of the chlorine based on experiment results.

## Result

### Corrosion kinetics

**Figure 1:**
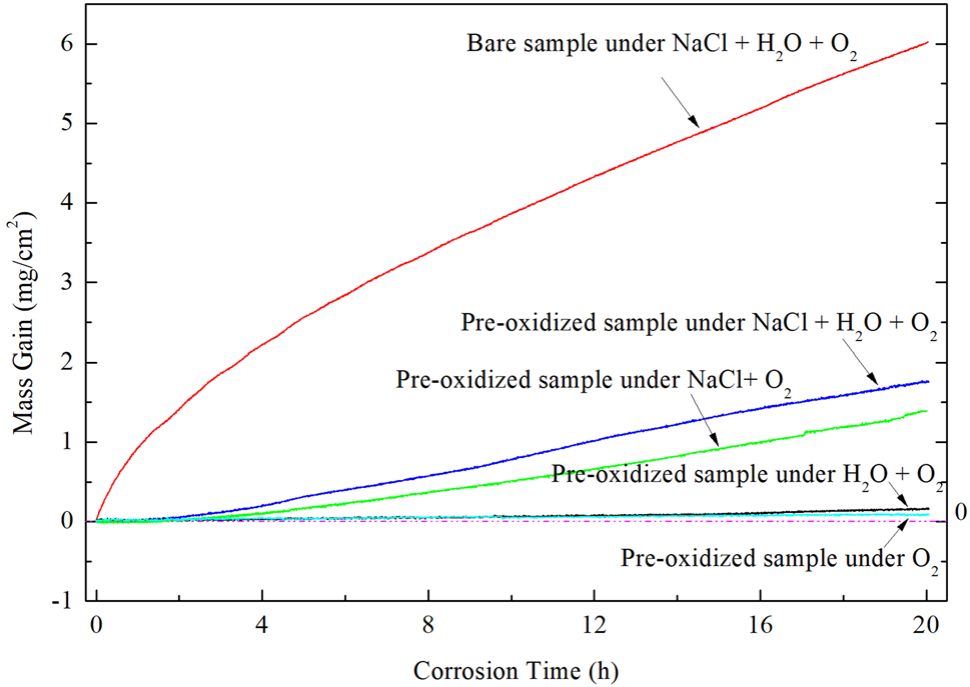
The mass gain curves for the corrosion at 600 ˚C of the bare Ti under NaCl + H_2_O + O_2_ (red line) and the pre-oxidized Ti under O_2_ (cyan line), H_2_O + O_2_ (black line), NaCl + O_2_ (green line) and NaCl + H_2_O + O_2_ (purple line).

The mass gain curves of pure Ti under different conditions are shown in Fig. 1. For the pre-oxidized samples, the mass gain under the condition of H_2_O + O_2_ (about 0.17 mg/cm^2^) is slightly larger than that under the condition of O_2_ (about 0.09 mg/cm^2^). In fact, under both the above two conditions the corrosion is minor. In the initial stage (incubation period), the mass gain is slight when the pre-oxidized pure Ti samples under NaCl deposit. In contrast, the mass gain increases rapidly after this incubation period. After 20 h exposure in NaCl + H_2_O + O_2_ or NaCl + O_2_, the mass gains are about 1.8 mg/cm^2^ and 1.4 mg/cm^2^, respectively, which are about one order of magnitude larger than that in the absence of the solid NaCl deposit. Thus, we reach the conclusion that the solid NaCl accelerates the corrosion of the pre-oxidized pure Ti in O_2_ or humid O_2_.

For the bare samples, the mass gain increases dramatically during the whole exposure in NaCl + H_2_O + O_2_. After exposed for 20 h, the mass gain is about 6.0 mg/cm^2^, which is increased by 3.5 times compared with the pre-oxidized samples. Therefore, we can conclude that the pre-oxidation improves the corrosion resistance of pure Ti coated with a solid NaCl deposit in humid O_2_ at 600 ˚C, especially in the incubation period.

### Phase composition and microstructure of the pre-oxidized sample

**Figure 2:**
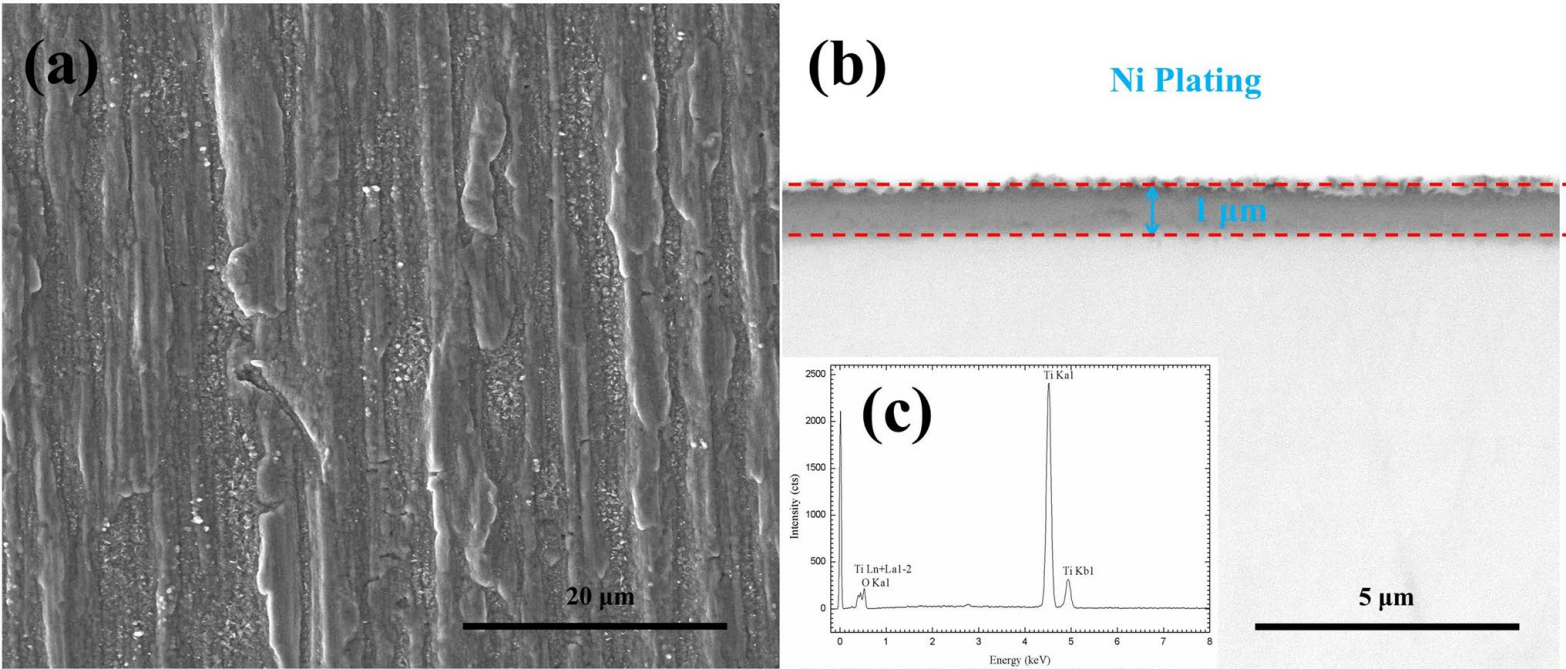
Surface morphologies (**a**), cross-sectional morphologies (**b**) and EDS patterns (**c**) of pure Ti oxidized in dry O_2_ at 600 ˚C for 20 h.

For the pure Ti samples after pre-oxidation in O_2_ flow for 20 h, the surface and the cross-sectional morphologies are shown in Fig. 2. As can be observed in Fig. 2a,b, a continuous, compact and even oxide scale is formed on the surface. The thickness of the oxide scale is about 1 μm (Fig. 2b). We show the results of EDS analysis on the oxide scale in Fig. 2c. As can be seen, it consists of Ti and O. Through XRD analysis (which is not shown here), the phase composition of the pre-oxidized sample surface is only TiO_2_.

### Phase composition and microstructure of the hot corrosion products forming on the bare samples

**Figure 3:**
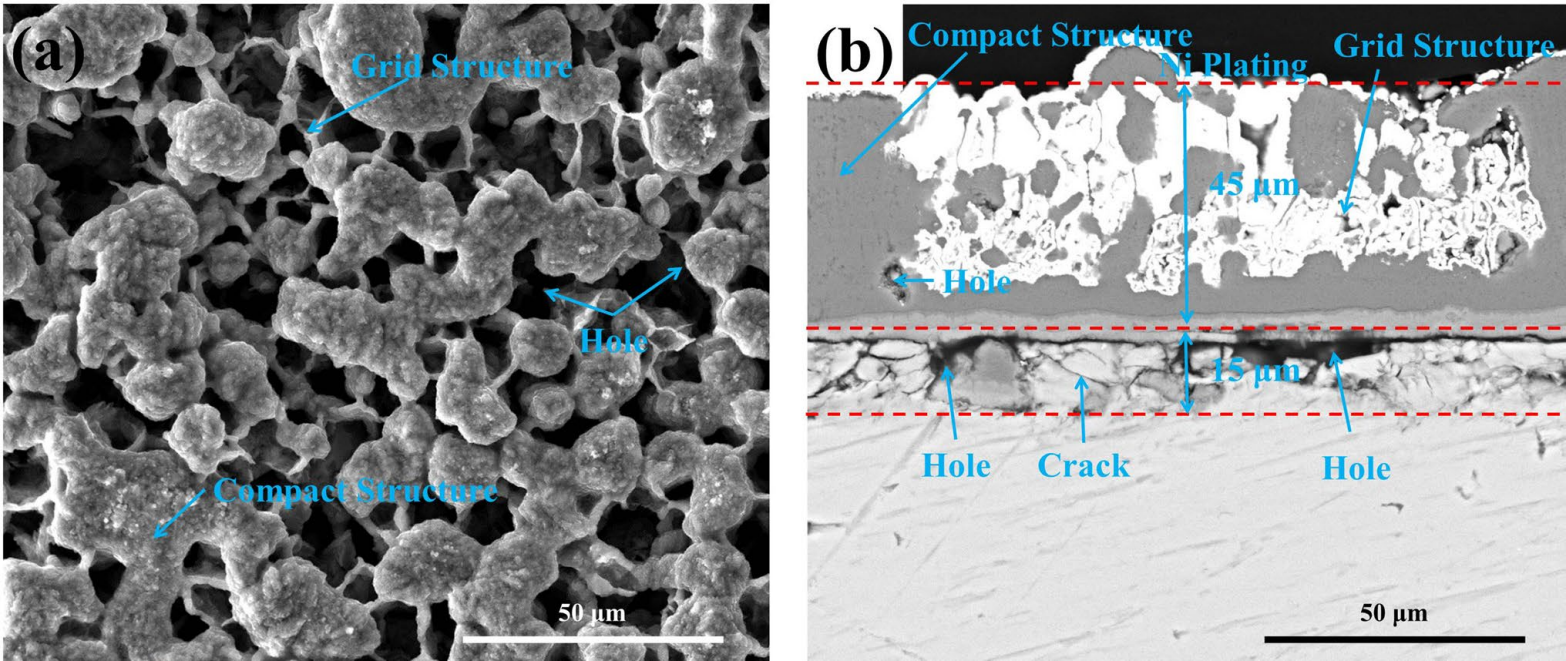
Surface morphologies (**a**) and cross-sectional morphologies (**b**) of the bare samples after corrosion in NaCl + H_2_O + O_2_ at 600 ˚C for 20 h.

For the bare samples after 20 h exposure in NaCl + H_2_O + O_2_, the surface and the cross-sectional morphologies are shown in Fig. 3. As can be observed, the scale of corrosion products contains two parts: one with a compact structure, and the other with a grid structure part. The corrosion products with a compact structure (marked in Fig. 3a,b) are very complete, uniform and compact. The corrosion products with a grid structure (marked in Fig. 3a,b) contain lots of holes (filled with Ni plating as shown in Fig. 3b), which penetrate the corrosion scale. These through-holes are the fast diffusion path for the corrosion medium. Below the corrosion scale, a loose layer, containing some holes (close pores) and cracks, is formed within the substrate due to the outward diffusion of Ti. The thickness of the corrosion product scale and the loose layer is about 45 μm and15 μm, respectively.

**Figure 4:**
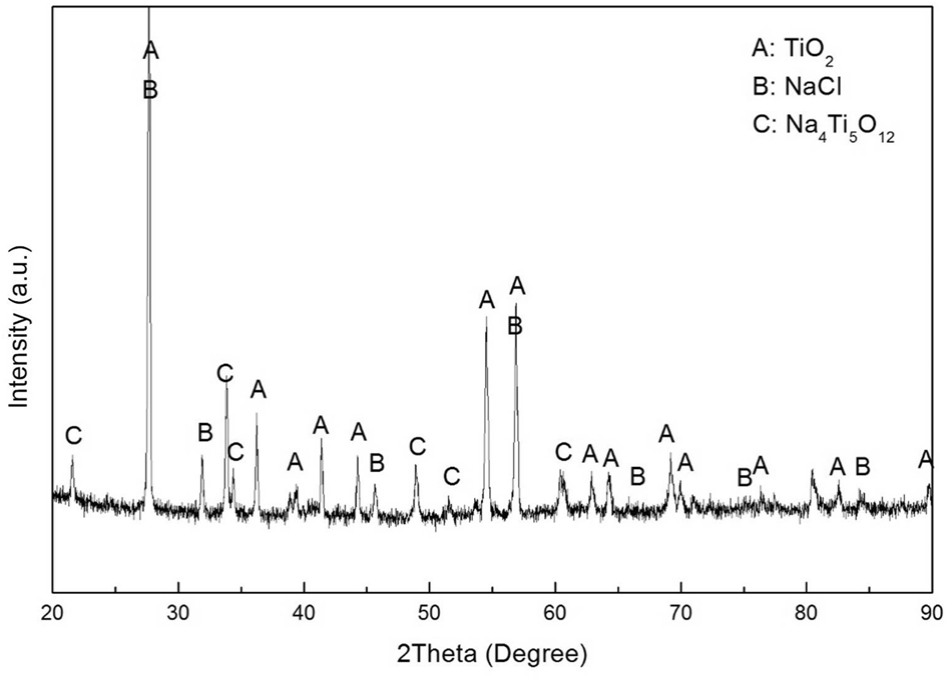
X-ray diffraction patterns of the bare samples exposed in NaCl + H_2_O + O_2_ up to 20 h at 600 ˚C.

In Fig. 4, we present the XRD patterns of the bare samples exposed in NaCl + H_2_O + O_2_ up to 20 h. The results show that the corrosion products mainly consist of TiO_2_ and some Na_4_Ti_5_O_12_, with the residual NaCl.

### Phase composition and microstructure of the hot corrosion products forming on the pre-oxidized samples

**Figure 5:**
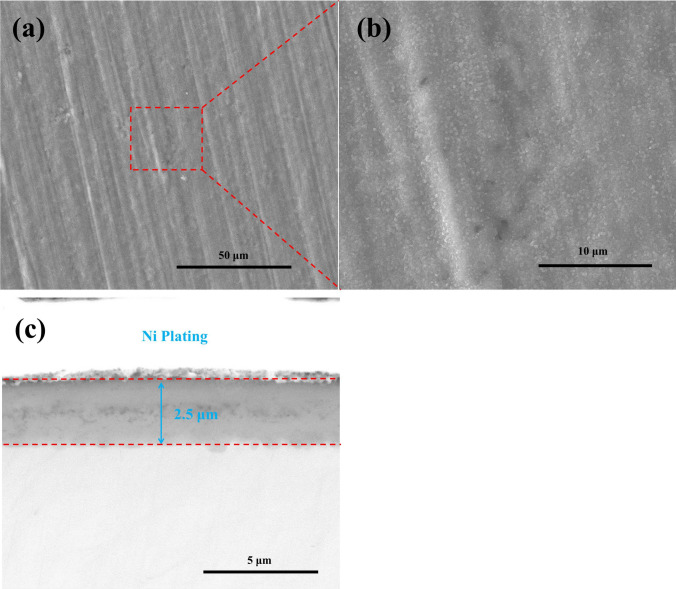
Surface morphologies (**a**), the high magnification image (**b**) and cross-sectional morphologies (**c**) of the pre-oxidized samples after oxidized in dry O_2_ at 600 ˚C for 100 h.

For the pre-oxidized pure Ti samples after 100 h exposure in dry O_2_ flow, the surface and the cross-sectional morphologies are shown in Fig. 5. As can be seen in Fig. 5a,b, a very thin, compact and continuous scale is formed on the pre-oxidized samples after 100 h exposure in dry O_2_ flow. From Fig. 5c, we can see that the thickness of the oxide scale is about 2.5 μm.

**Figure 6:**
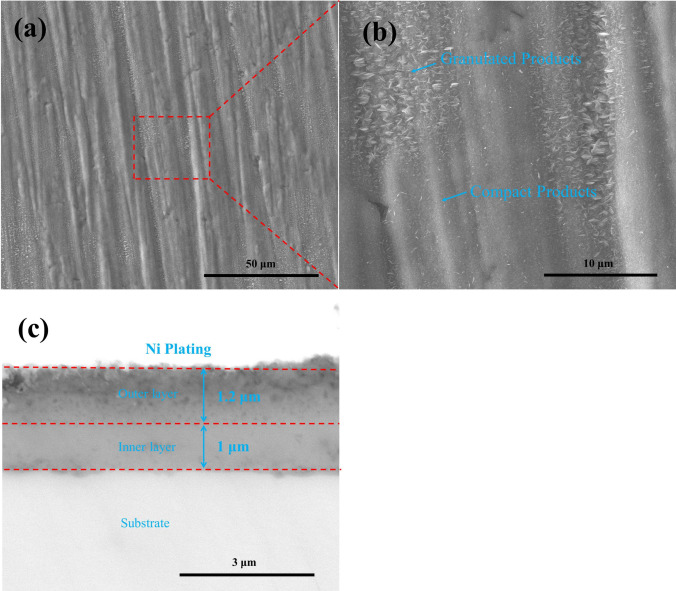
Surface morphologies (**a**), the high magnification image (**b**) and cross-sectional morphologies (**c**) of the pre-oxidized samples after oxidized in humid O_2_ at 600 ˚C for 100 h.

For the pre-oxidized samples after 100 h exposure in humid O_2_ flow, the surface and the cross-sectional morphologies are presented in Fig. 6. As can be observed from Fig. 6a,b, a very thin, compact and continuous scale which contains both granulated corrosion products and compact products was formed on the pre-oxidized samples. From Fig. 6c, we can see that the oxide scale is divided into a loose outer layer and a compact inner layer, with a thickness of about 1.2 μm and 1 μm, respectively.

**Figure 7:**
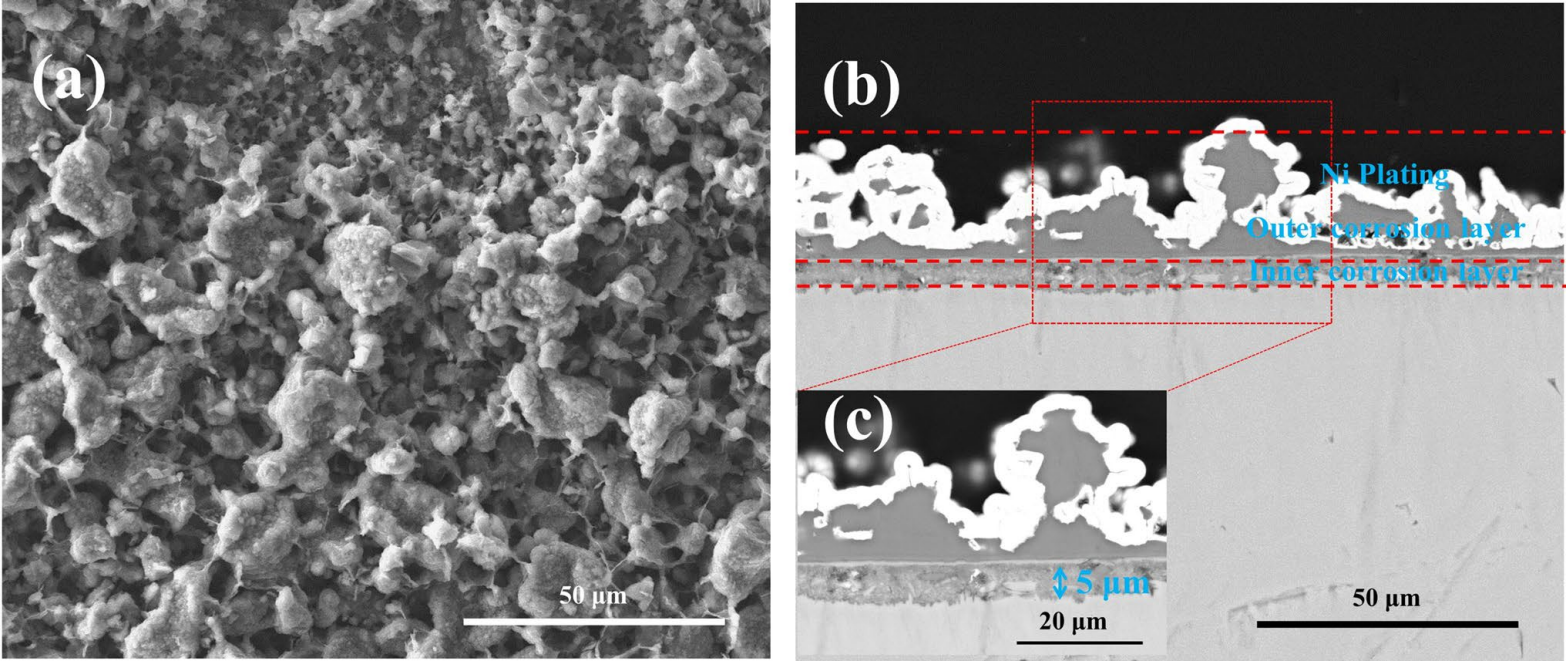
Surface morphologies (**a**) and cross-sectional morphologies (**b**) and the high magnification image (**c**) of the pre-oxidized samples after corrosion in NaCl + O_2_ at 600 ˚C for 20 h.

For the pre-oxidized samples after 20 h exposure in NaCl + O_2_, the surface and the cross-sectional morphologies are displayed in Fig. 7. As can be seen in Fig. 7a, the corrosion products on the surface are in clusters. As can be observed in Fig. 7b,c, the corrosion product scale can be divided into an outer layer and an inner layer. The outer layer is filled with clustered corrosion products, and the inner layer whose thickness is about 5 μm contains loose corrosion products.

**Figure 8:**
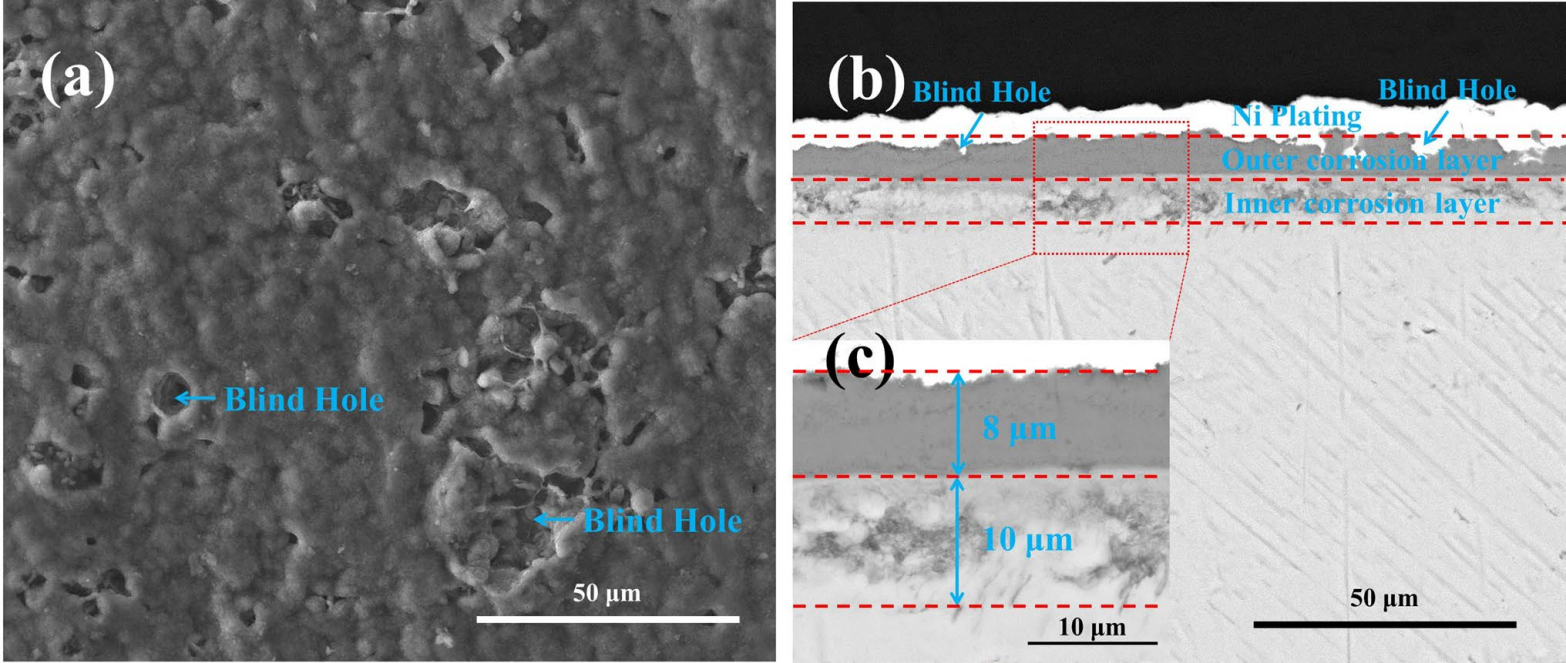
Surface morphologies (**a**) and cross-sectional morphologies (**b**) and the high magnification image (**c**) of the pre-oxidized samples after corrosion in NaCl + H_2_O + O_2_ at 600 ˚C for 20 h.

For the pre-oxidized samples after 20 h exposure in NaCl + H_2_O + O_2_, the surface and the cross-sectional morphologies are depicted in Fig. 8. As can be observed in Fig. 8a,b, corrosion products have a very thin, compact and continuous scale, with some blind holes forming on the surface where we can also observe some defects (like pore). Furthermore, the scale can be divided into a loose inner corrosion layer and a compact outer corrosion layer. As shown in Fig. 8c, the thickness of the outer corrosion layer and the inner corrosion layer is 8 μm and 10 μm, respectively.

**Figure 9:**
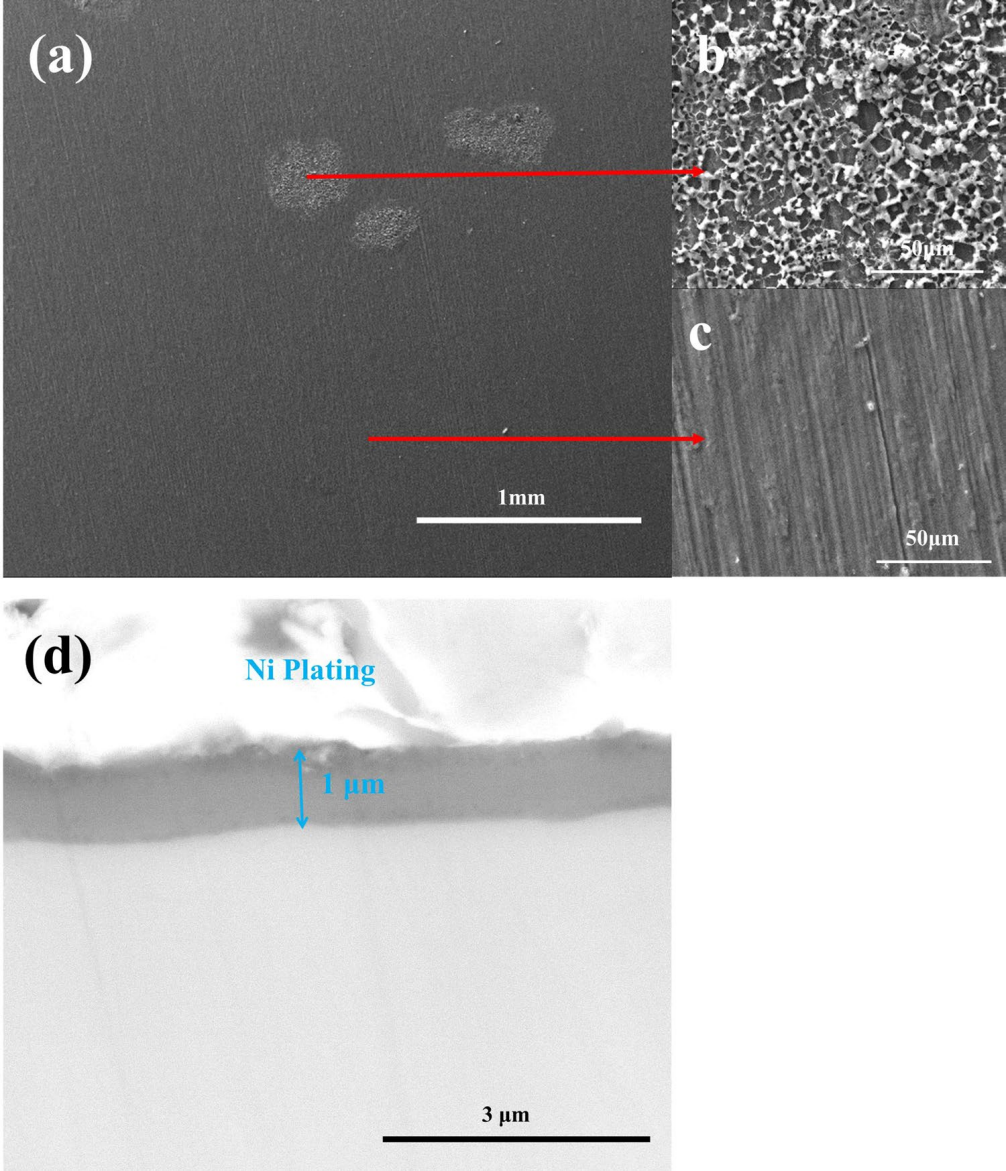
Surface morphologies (**a**), the high magnification image (**b, c**) and cross-sectional morphologies (**d**) of the pre-oxidized samples under a solid NaCl deposit layer in humid O_2_ for 1 h at 600 ˚C.

For the pre-oxidized samples after 1 h exposure in NaCl + H_2_O + O_2_, the surface and the cross-sectional morphologies are shown in Fig. 9. From Fig. 9a, we can observe a compact scale on which some loose corrosion products are scattered. In Fig. 9b, we can observe that under the loose product, the scale still remains complete and compact. This implies that the loose products are formed due to the reaction of the outward diffusion metal ions with the corrosive species. From Fig. 9d, we can observe that the thickness of this scale is about 1 μm.

**Figure 10:**
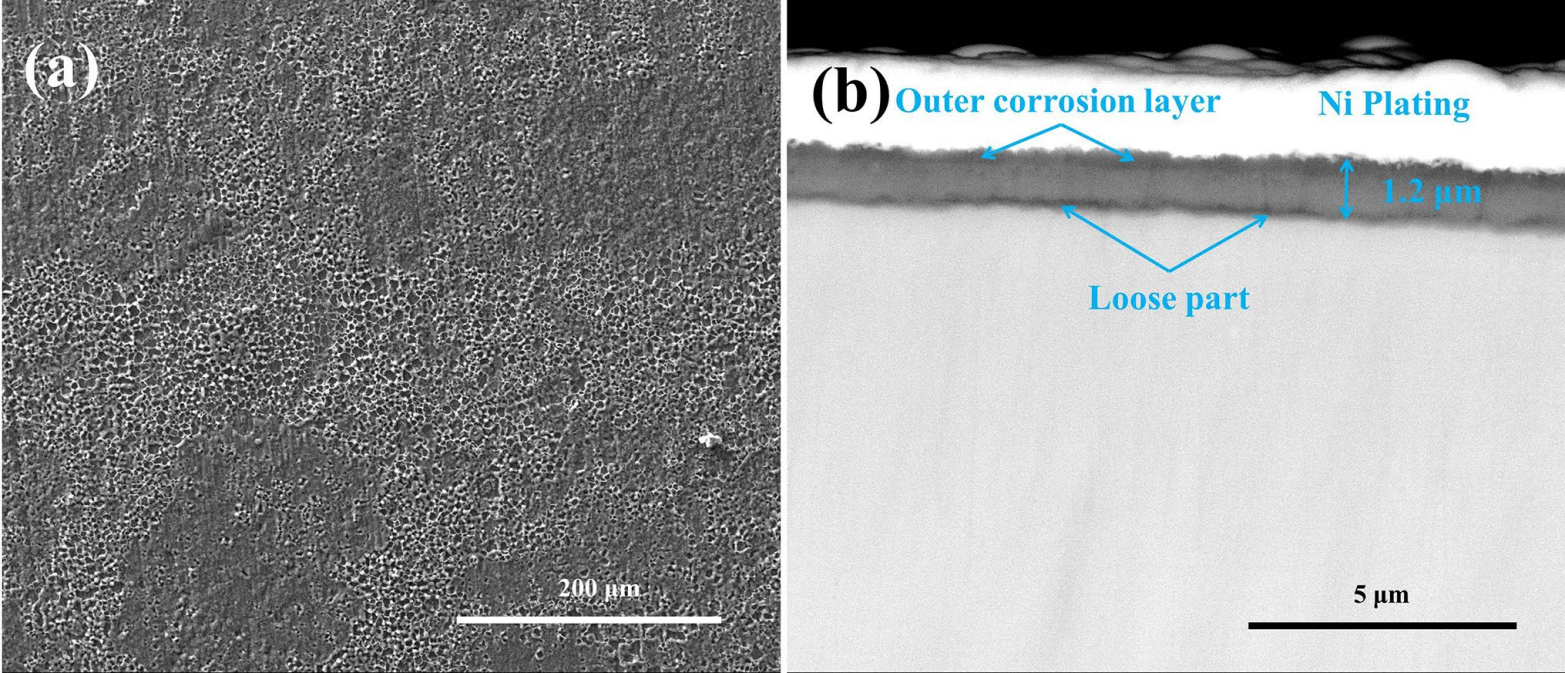
Surface morphologies (**a**) and cross-sectional morphologies (**b**) of the pre-oxidized samples under a solid NaCl deposit layer in humid O_2_ for 5 h at 600 ˚C.

For the pre-oxidized samples after 5 h exposure in NaCl + H_2_O + O_2_, the surface and the cross-sectional morphologies are depicted in Fig. 10. From Fig. 10a, we can observe that the porous corrosion products spread over the entire surface. This is consistent with the fact in Fig. 1 that the mass gain increased rapidly after the incubation period and the corrosion is severe. From Fig. 10b, we can observe that the scale of the corrosion products is compact and its thickness is about 1.2 μm. We can also observe some loose parts at the interface between the scale and metal.

**Figure 11:**
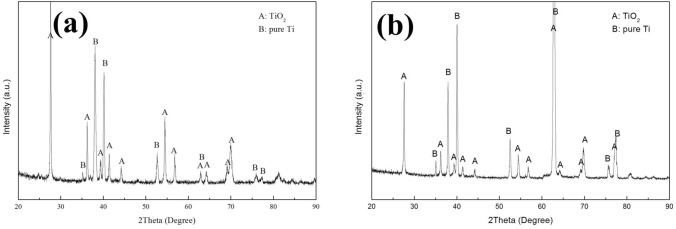
X-ray diffraction patterns of the pre-oxidized samples after exposed in dry O_2_ flow (**a**) and in humid O_2_ flow (**b**) for 100 h.

**Figure 12:**
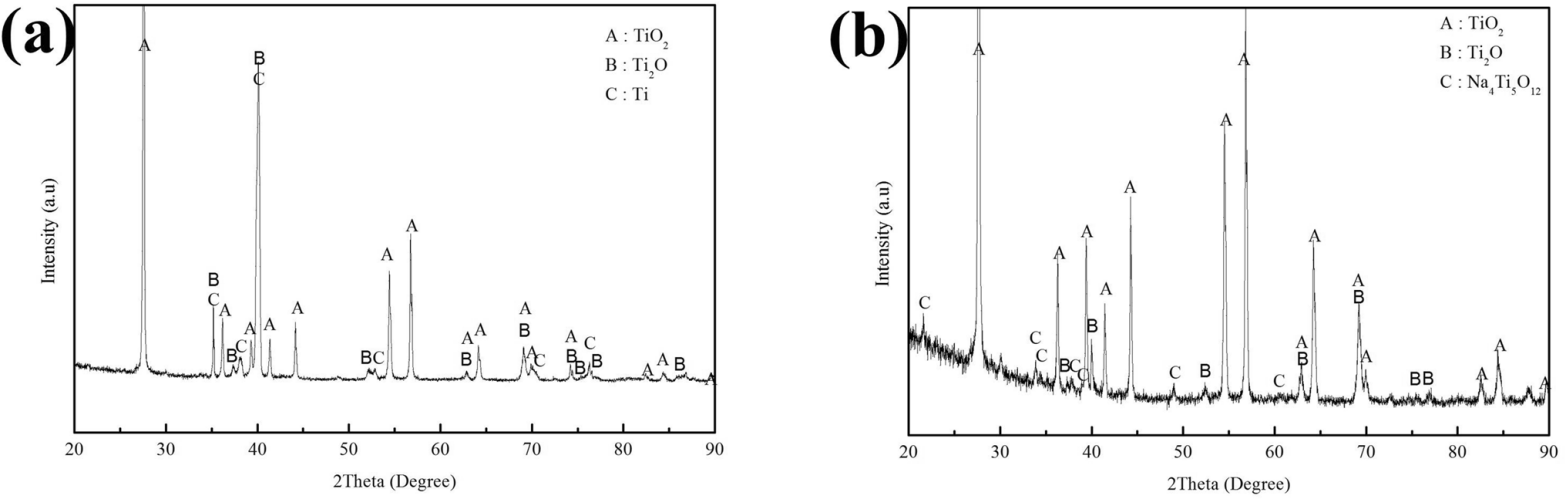
X-ray diffraction patterns of the pre-oxidized samples after exposed in NaCl + O_2_ for 5 h (**a**) and for 20 h (**b**).

**Figure 13:**
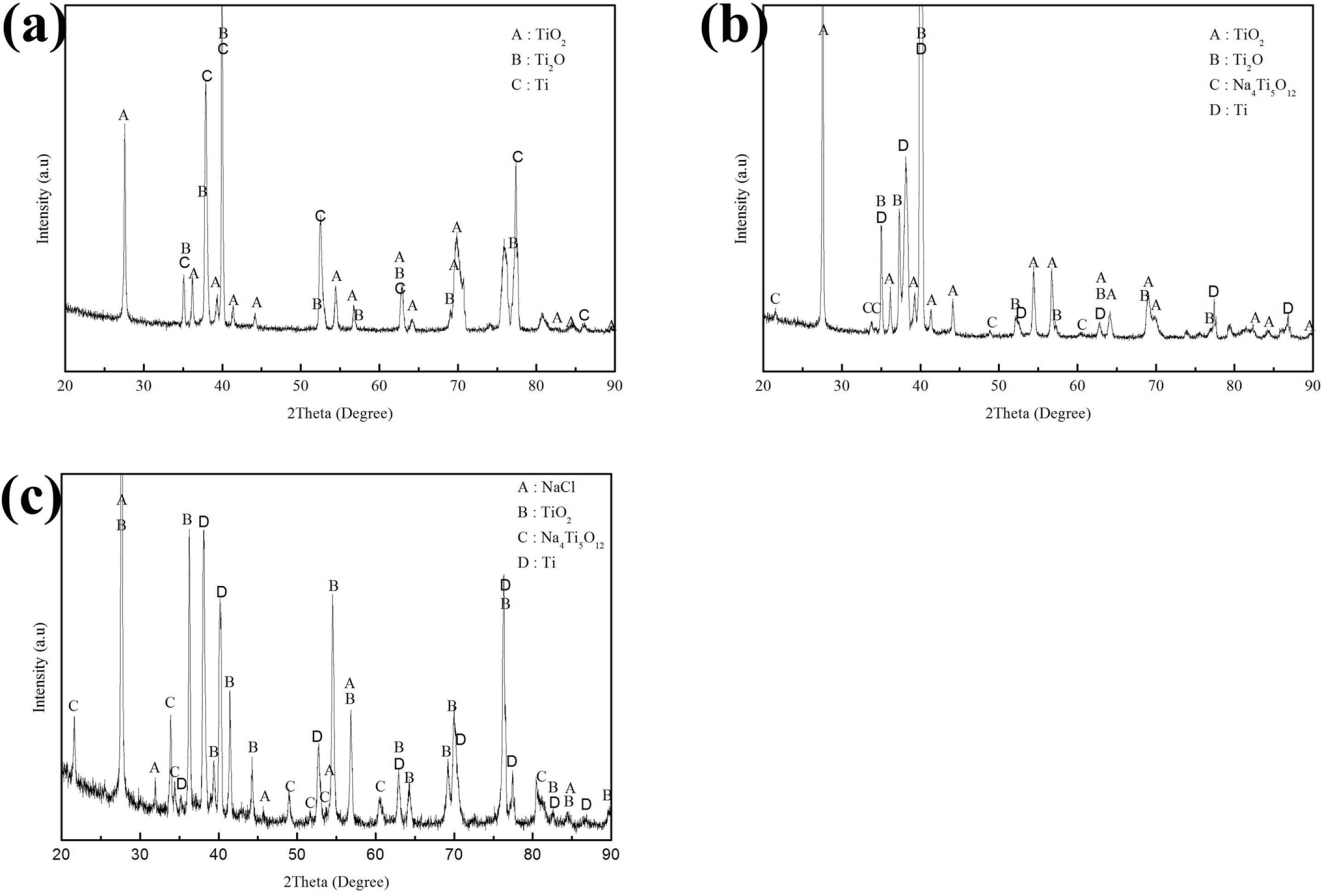
X-ray diffraction patterns of the pre-oxidized samples after exposed in NaCl + H_2_O + O_2_ for 1 h (**a**), 5 h (**b**) and 20 h (**c**).

The corrosion products of the pure Ti samples are identified by XRD. The XRD patterns in Fig. 11 show that only TiO_2_ was formed on the pre-oxidized samples after 100 h exposure in dry O_2_ flow (Fig. 11a) or in H_2_O + O_2_ flow (Fig. 11b). In Fig. 12, we can observe that the corrosion products are TiO_2_, Ti_2_O and Na_4_Ti_5_O_12_ on the pre-oxidized samples after 20 h exposure in NaCl + O_2_ (Fig. 12b), whereas the Na_4_Ti_5_O_12_ cannot be identified by XRD after 5 h exposure (Fig. 12a). As shown in Fig. 13c, after the pre-oxidized samples are exposed in NaCl + H_2_O + O_2_ for 20 h, the corrosion products contain both TiO_2_ and Na_4_Ti_5_O_12_. There still remains residual NaCl as well as the substrate (pure Ti) on the surface. As shown in Fig. 13b, after 5 h exposure in NaCl + H_2_O + O_2_ (which is after the incubation period), the corrosion products also contain Na_4_Ti_5_O_12_. Since the diffracted intensity of Na_4_Ti_5_O_12_ in Fig. 13c are larger than that in Fig. 13b, we can infer that more Na_4_Ti_5_O_12_ is formed when the pre-oxidized samples are exposed in NaCl + H_2_O + O_2_ for a longer time period. As shown in Fig. 13a, after 1 h exposure in NaCl + H_2_O + O_2_ (which is during the incubation period), there are not Na_4_Ti_5_O_12_ among the corrosion products. We conclude the corrosion products under the above different conditions in Table 1.

**Table 1:** Corrosion products forming on the bare samples and the pre-oxidized samples after hot corrosion.

Samples	Environment	Corrosion time			
		1 h	5 h	20 h	100 h
Bare samples	NaCl + H_2_O + O_2_	–	–	TiO_2_, Na_4_Ti_5_O_12_	–
Pre-oxidized samples	NaCl + H_2_O + O_2_	TiO_2_, Ti_2_O	TiO_2_, Ti_2_O, Na_4_Ti_5_O_12_	TiO_2_, Ti_2_O, Na_4_Ti_5_O_12_	–
	NaCl + O_2_	–	TiO_2_, Ti_2_O	TiO_2_, Ti_2_O, Na_4_Ti_5_O_12_	–
	H_2_O + O_2_	–	–	–	TiO_2_
	O_2_	–	–	–	TiO_2_

### Time of flight-secondary ion mass spectrometry, ToF–SIMS

**Figure 14:**
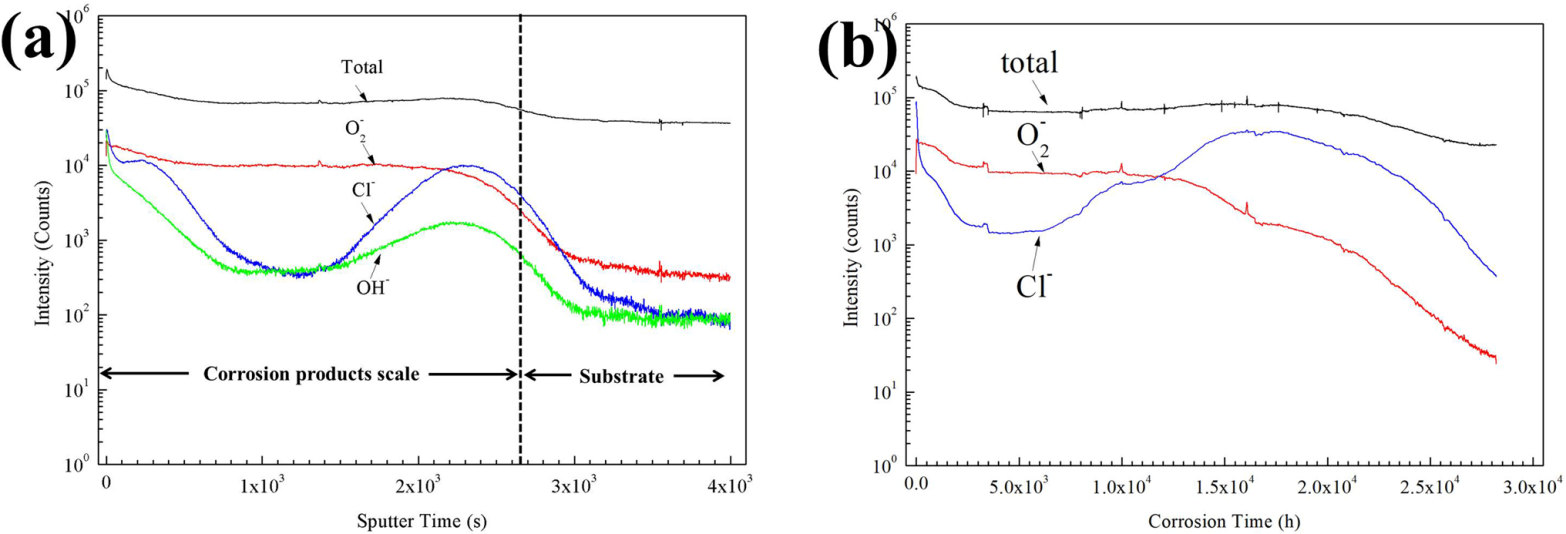
ToF–SIMS sputter depth profiles in the corrosion products scales formed on the pre-oxidized samples under a NaCl deposit in an atmosphere of humid O_2_ at 600 ˚C for 1 h (**a**) and 5 (**b**).

Figure 14 shows the depth profiles for the negative ions from the pre-oxidized samples after 1 h and 5 h exposure in NaCl + H_2_O + O_2_. The ${\text{O}}_{2}^{ - }$ ion signal in Fig. 14 is applied here to indicate the distribution of oxygen in the scale and to identify the interface between the corrosion products scale and the substrate. In Fig. 14a, the ${\text{O}}_{2}^{ - }$ ion exhibits two levels of concentration, and the transition region is around the interface of the corrosion product scale and the substrate. Therefore, the average of logarithmic value is pinpointed as the interface as shown in Fig. 14a. Similarly, this interface is also identified in Fig. 14b.

In Fig. 14a, we can also observe that after 1 h exposure in NaCl + H_2_O + O_2_, Cl^-^ ions first enrich on the surface of the pre-oxidized samples (which was caused by the residual NaCl) and then decrease rapidly in the corrosion product scale. Afterwards, Cl^-^ is enriched at the oxide/metal interface, which indicates that the chlorine penetrates the oxide and accumulates at the oxide/metal interface. In Fig. 14b, after the pre-oxidized samples are exposed in NaCl + H_2_O + O_2_ for 5 h, a similar distribution of Cl^-^ in the corrosion product scale was observed. Particularly, the Cl^-^ ion is still enriched when the ${\text{O}}_{2}^{ - }$ ion decreases around the oxide/metal interface, which implies that some chlorine diffuses into the metal substrate. The intensity of Cl^-^ around the oxide/metal interface is stronger in Fig. 14b than that in Fig. 14a. This implies that more chlorine penetrates into the oxide after a longer exposure in the presence of solid NaCl.

The OH^-^ signal in Fig. 14 is applied here to indicate the distribution of H in the scale. From Fig. 14a, the distribution of the OH^-^ is similar with Cl^-^. First, the OH^-^ ion enriches on the surface and then decrease rapidly in the corrosion product scale. This is the information of water vapor. Afterwards, OH^-^ is enriched at the oxide/metal interface where Cl^-^ is accumulated, which indicates that the H penetrates the oxide along with the chlorine.

## Discussion

### The acceleration of NaCl to the corrosion of pure Ti

As shown in Fig. 2, after pure Ti samples were oxidized in the pure O_2_ flow at 600 ˚C for 20 h, a compact TiO_2_ scale with a thickness of about 1 μm was formed on the surface, which is caused by the following reaction between the metal substrate and O_2_:

1 $$Ti + O_{2} = TiO_{2}$$

In contrary, the mass gain of pure Ti under a solid NaCl deposit layer (NaCl + H_2_O + O_2_) increased quickly and the mass gain is about 6.0 mg/cm^2^ after 20 h exposure (Fig. 1), which is much larger than that in the absence of solid NaCl (about 0.3 mg/cm^2 10^). The resulting scale consists of plentiful and porous corrosion products (Fig. 3) and its thickness is about 45 μm. The above phenomena indicate that pure Ti suffered severe corrosion when exposed in NaCl + H_2_O + O_2_. This severe corrosion has been discussed in our previous work^10^ and the main reason is that the protective TiO_2_ scale cannot be formed due to the occurrence of a series of chemical reactions, and the corrosion becomes active. In the previous mechanism, NaCl first destroys the protective scale as follow:

2 $$5TiO_{2} + 4NaCl + 2H_{2} {\text{O}} = Na_{4} Ti_{5} O_{12} + 4HCl$$

3 $$6TiO_{2} + 4NaCl = Na_{4} Ti_{5} O_{12} + TiCl_{4}$$

The TiCl_4_ reacts with H_2_O to form HCl:

4 $$TiCl_{4} + 2H_{2} O = TiO_{2} + 4HCl$$

Then, HCl reacts with the substrate cyclically:

5 $$2HCl + Ti = TiCl_{2} + H_{2}$$

6 $$4HCl + Ti = TiCl_{4} + 2H_{2}$$

7 $$2TiCl_{2} + O_{2} + 2H_{2} O = 2TiO_{2} + 4HCl$$

8 $$2H_{2} { + }O_{2} = 2H_{2} O$$

These reactions are thermodynamically spontaneous due to the negative ΔGº at 600 ˚C, as presented in Table 2.

As shown in Fig. 3b, in the corrosion product scale there are holes which provide rapid diffusion channels for corrosive species (for example, oxygen, chlorine and water vapor). Meanwhile, a corrosion scale is formed by the outward diffusion of Ti which results in a 15 μm loose layer that is observed under the corrosion products. Hence, the corrosion product scale is non-protective and the corrosion of pure Ti is greatly accelerated by solid NaCl in an O_2_ + H_2_O environment at 600 ˚C, which is similar with the observation for several Ti alloy^11,18–20^.

### The protection of pre-oxidation scale to the pure Ti

After pre-oxidation in O_2_ flow for 20 h, a continuous, compact and even oxide scale is formed on the surface (Fig. 2). The pre-oxidation scale is only TiO_2_ (Fig. 2c) and its thickness is about 1 μm (Fig. 2b). Since this scale can protect the substrate, the mass gain of the pre-oxidized samples is small in the dry O_2_ or humid O_2_ (Fig. 1).

When pre-oxidized samples are exposed in the presence of solid NaCl (NaCl + O_2_ and NaCl + H_2_O + O_2_), the mass gain of the pre-oxidized samples is smaller than that of the bare samples (Fig. 1), especially in the incubation period (the mass gain increases slowly). The resulting scale on the pre-oxidized samples (which is compact as shown in Figs. 7 and 8) is thinner than that on the bare samples (which is porous as shown in Fig. 3). Thus, the pre-oxidation can protect the pure Ti samples from the corrosion under NaCl deposit. This is mainly attributed to the compact TiO_2_ scale formed on the surface in the pre-oxidation (Fig. 2). During the pre-oxidation, the thickness of the formed TiO_2_ scale is about 1 μm, and the pre-oxidation scale is larger thicker than the passive film forming on the bare sample. Thus, the TiO_2_ scale can be a barrier layer for the diffusion of corrosive species (for example, oxygen, water vapor and HCl) and reduce the corrosion to a certain extent, especially in the incubation period.

### The destruction of pre-oxidation scale

When exposed in humid O_2_, the pre-oxidized samples suffer a slightly more serious oxidation than when exposed in dry O_2_ (Fig. 1). This is because H_2_O tends to dissociate at the defects on TiO_2_/(110) planes into free H atoms and OH^-^ groups^21–23^ when the pre-oxidized samples is exposed in humid O_2_. The papers^21,22^ reported that along the crystal channels in c-axis direction, the diffusion rate of hydrogen is at least one order of magnitude larger than that in the perpendicular direction of c-axis. Thus, the generated hydrogen atoms are more prone to diffuse into TiO_2_ through the former one. This dissolution of hydrogen in TiO_2_ will form hydrogen defects, thus the hydrogen can increase the concentration of crystal defects and increase the outward diffusion of Ti ions^23^. This fast diffusion in the scale causes a larger corrosion rate and hence the hole forms in the oxide scale (Fig. 6), which make the corrosion product scale thicker and more porous (Fig. 6c). Thus, the water vapor slightly reduces the protection of the pre-oxidation scale by forming the H defects.

From Fig. 1, we can observe the following: (1) after the incubation period, the mass gain of the pre-oxidized samples increases more rapidly when exposed in the presence of the solid NaCl (NaCl + O_2_ or NaCl + H_2_O + O_2_) than when exposed in the absence of the solid NaCl (O_2_ or H_2_O + O_2_); (2) after 20 h exposure, the pre-oxidized samples has a larger mass gain under the condition of NaCl + O_2_ or NaCl + H_2_O + O_2_ than under the condition of O_2_ or H_2_O + O_2_. Additionally, we can observe that the corrosion product scale of the pre-oxidized samples is thicker when exposed in the presence of the solid NaCl (Figs. 7 and 8) than when exposed in the absence of the solid NaCl (Figs. 5 and 6). Thus, we can claim that the corrosion of the pre-oxidized samples is accelerated by solid NaCl. The fast corrosion and the formation of Na_4_Ti_5_O_12_ on the surface (Fig. 9c,d) indicate that the “protective oxide” formed in the pre-oxidation is not completely inert to solid NaCl.

### The diffusion of the chlorine in the pre-oxidation scale

The evidence from XRD and SEM/EDX show that Na_4_Ti_5_O_12_ formed on the surface (see Figs. 4 and 9c,d). This indicated that the solid NaCl reacted with the TiO_2_ scale. Similar observations have been reported for Ti alloy using the same experimental conditions^18^. In that case, it was argued that the fast corrosion in an O_2_ + H_2_O environment is triggered by titanate formation. Thus, in an O_2_ + H_2_O + NaCl environment, the formation of titanate destroys the protective scale formed on the surface, and then the gathered chlorine circularly reacts with the substrate. Thus, for the corrosion of pure Ti/Ti alloy in the NaCl + H_2_O + O_2_ environment, the chlorine is the main factor.

For the pre-oxidized samples, a thick TiO_2_ scale is formed on the surface; thus, it takes more time to destroy the protective TiO_2_ scale by Na_4_Ti_5_O_12_ formation and the mass gain changes little during the incubation period (Fig. 1). Furthermore, after the sodium forms Na_4_Ti_5_O_12_, the gathered chlorine diffuses inward to the metal/oxide interface (Fig. 14a). Shu et al. ^18^ report that the chlorine permeates into the corrosion product scale as molecule, like HCl or Cl_2_. For the pre-oxidized samples, a compact TiO_2_ formed on the surface protects the substrate from the inward diffusion of chlorine and the fast corrosion. Nevertheless, during the incubation period, the chlorine also diffuses into the metal/oxide interface (Fig. 14a) while the TiO_2_ scale is complete (Fig. 9). This implies that the chlorine cannot be molecule. In our previous work, the chlorine forms Ti-Cl bond in the inner corrosion layer by the dopant Cl replacing O in the Ti oxides lattice when Ti60 alloy was exposed in the NaCl + H_2_O + O_2_. On the other hand, the diffusion of ion in the TiO_2_ is fast and some NaCl is split into ions (Na^+^ and Cl^-^) at 600 ˚C. Hence, we think the chlorine permeates into the corrosion product scale as ion (Cl^-^).

As shown in Fig. 14b, when the pre-oxidized samples suffer from severe corrosion in NaCl + H_2_O + O_2_, the chlorine is also observed around the metal/oxide interface by SIMS. Additionally, the intensity of chlorine is still large when the intensity of oxygen decreases to a relatively small value. As aforementioned, the substrate locates at the places where the intensity of oxygen is small. Hence, we can infer that the chlorine diffuses into the substrate and directly binds with the metal of the substrate during the fast corrosion. The gathered compound diffuses outward, resulting in the loss of metal in the substrate. After 20 h exposure, a mass of the metal reacts with the chlorine and diffuses outward, and the inner corrosion layer is formed under the outer corrosion layer (Fig. 8b).

## Conclusion

During the pre-oxidation, a compact and thick TiO_2_ scale forms on the surface of pure Ti. This TiO_2_ scale is the barrier layer for the diffusion of corrosive species (for example, oxygen, chlorine, and water vapor), which can protect the substrate from fast corrosion. However, the “protective oxide” is not completely inert to solid NaCl and the corrosion rate of the pre-oxidized sample is also greatly accelerated by solid NaCl after an incubation period. During the incubation period, the chlorine, generated in the decomposition of solid NaCl at 600 ˚C, diffuses into the oxide/substrate interface as ions to enhance the corrosion rate, and then the residual sodium reacts with the oxide on the surface. After the incubation period, the chlorine diffuses into the substrate and directly reacts with the substrate, resulting in the fast corrosion.

## Experiment

### Materials preparation

The material used in this study was pure Ti and the samples were cut into the size of 10 mm × 15 mm × 2.5 mm. Prior to experiments, all samples were mechanically grinded with 800# SiC paper, ultrasonically degreased in alcohol for about 20 min, and dried in the air. The bare samples were then exposed in the pure O_2_ flow at 600 ˚C for 20 h for the pre-oxidization. The surfaces of preheated samples were covered with a layer of NaCl deposit by repeatedly brushing and drying with saturated NaCl solution^1,6,8,9,18,24^, until about 4 ± 0.2 mg/cm^2^ solid NaCl was deposited on.

### Corrosion experiments

The corrosion tests were carried out in a thermo-balance ^1,6,8,9,24^. The continuous mass gain during the corrosion experiment was obtained with a thermo-gravimetric analysis (TGA).

The pure O_2_ was bubbled into the distilled water with a glass bubbler (when the inner diameter of the tube was about 3.2 cm, the flow rate of pure O_2_ was about 140 mL/min in this study) to produce the test atmosphere (humid O_2_). According to the relationship between the vapor pressure of water and its temperature, we precisely set up the temperature of the distilled water in the glass bubbler to control the amount of the water vapor. In this study, the temperature of the distilled water was about 70 ˚C, producing about 30.8 vol.% water vapor. In order to avoid the water vapor condensing inside the thermo-balance, a counter flow of pure N_2_ was passed through the thermo-balance, whose flow rate was about 400 mL/min. After the furnace reached 600 ˚C and the gas flows of humid O_2_ as well as pure N_2_ stabilized, the samples were quickly lowered into the constant temperature zone of the furnace tube.

In this study, the corrosion experiments were respectively carried out under the following four conditions: the first condition was with a solid NaCl deposit layer in a humid O_2_ flow at 600 ˚C (denoted as NaCl + H_2_O + O_2_); the second condition was carried out in a humid O_2_ flow at 600 ˚C (denoted as H_2_O + O_2_); the third condition was with a solid NaCl deposit layer in a dry O_2_ flow at 600 ˚C (denoted as NaCl + O_2_); and the fourth condition was carried out in a dry O_2_ flow at 600 ˚C (denoted as O_2_). We present the explicit environmental parameters in Table 3.

**Table 2:** Standard Gibbs free energy changes of reactions at 600 ˚C.

Reaction	ΔGº * kJ/mol
Ti + O_2_ → TiO_2_	− 785
4NaCl + 6TiO_2_ → Na_4_Ti_5_O_12_ + TiCl_4_	− 65
4NaCl + 5TiO_2_ + 2H_2_O → Na_4_Ti_5_O_12_ + 4HCl	− 195
TiC_4_ + 2H_2_O →TiO_2_ + 4HCl	− 130
Ti + 2HCl → TiCl_2_ + H_2_	− 174
Ti + 4HCl → TiCl_4_ + 2H_2_	− 256
2TiCl_2_ + 2H_2_O + O_2_ → 2TiO_2_ + 4HCl	− 905
2H_2_+O_2_ → 2H_2_O	− 399

* The Gº value of Na_4_Ti_5_O_12_ was calculated by the first principles. And others were calculated by the HSC Chemistry.

**Table 3:** Experimental parameters.

Environment	Mass of NaCl (mg/cm^2^)	Water pressure (kPa)	Flow rate of O_2_ (mL/min)	Temperature (˚C)
NaCl + H_2_O + O_2_	4.0	31	140	600
NaCl + O_2_	4.0	0	140	600
H_2_O + O_2_	0	31	140	600
O_2_	0	31	140	600

### Morphologies and chemical composition analysis

The surface morphologies and the cross-sectional morphologies of corrosion products scale were collected by SEM–EDS. The chemical composition of corrosion products was identified by XRD.

To protect the oxide scale from fracture and spall during the metallographic preparation, the corroded samples were wrapped into a thin nickel foil by electroless plating and were embedded into the epoxy resin. Then, the corroded samples were grinded to 3000 grit with SiC paper. Finally, the corroded samples were polished with diamond paste. The samples were washed with distilled water to remove the residual solid NaCl, and then dried in air, before surface investigation by SEM. Under the condition of H_2_O + O_2_ and O_2_, the mass gains of the pre-oxidized sample were very small (Fig. 1) and the corrosion was minor. The surface and the cross-sectional morphologies after a longer corrosion time (100 h) were investigated.

### Time of flight-secondary ion mass spectrometry, ToF–SIMS

The pre-oxidized samples were first exposed in either NaCl + H_2_O + O_2_ or NaCl + O_2_. Then, they are ultrasonically washed with distilled water to remove the residual solid NaCl. After the pre-oxidized samples were further dried in the air, they were analyzed using a ToF-SIMS5 instrument (ION-TOF GmbH), which allowed parallel mass registration with high sensitivity and high mass resolution. A cesium liquid–metal ion (LMI) gun at 20 keV beam energy was used for spatially resolved ToF–SIMS analysis.

## References

[1] Shu Y, Wang F, Wu W (2000). Corrosion behavior of pure Cr with a solid NaCl deposit in O_2_ plus water vapor. Oxid. Met..

[2] Liu L, Li Y, Zeng C, Wang F (2006). Electrochemical impedance spectroscopy (EIS) studies of the corrosion of pure Fe and Cr at 600 °C under solid NaCl deposit in water vapor. Electrochim. Acta.

[3] Tang Y, Liu L, Li Y, Wang F (2011). The electrochemical corrosion mechanisms of pure Cr with NaCl deposit in water vapor at 600 °C. J. Electrochem. Soc..

[4] Tang Y, Liu L, Li Y, Wang F (2010). Evidence for the occurrence of electrochemical reactions and their interaction with chemical reactions during the corrosion of pure Fe with solid NaCl deposit in water vapor at 600 °C. Electrochem. Commun..

[5] Tang Y, Liu L, Fan L, Li Y, Wang F (2014). The corrosion behavior of pure iron under solid Na_2_SO_4_ deposit in wet oxygen flow at 500 °C. Materials.

[6] Wang F, Shu Y (2003). Influence of Cr content on the corrosion of Fe-Cr alloys: the synergistic effect of NaCl and water vapor. Oxid. Met..

[7] 7Pujilaksono, B. *et al.* Oxidation of binary FeCr alloys (Fe–2.25Cr, Fe–10Cr, Fe–18Cr and Fe–25Cr) in O_2_ and in O_2_ + H_2_O environment at 600 °C. *Oxidation of Metals* **75**, 183–207, 10.1007/s11085-010-9229-z (2011).

[8] Shu Y, Wang F, Wu W (1999). Synergistic effect of NaCl and water vapor on the corrosion of 1Cr-11Ni-2W-2Mo-V steel at 500–700 ˚C. Oxid. Met..

[9] Wang F, Geng S, Zhu S (2002). Corrosion behavior of a sputtered K38G nanocrystalline coating with a solid NaCl deposit in wet oxygen at 600 to 700 ˚C. Oxid. Met..

[10] Fan L (2016). Corrosion behavior of pure Ti under a solid NaCl deposit in a wet oxygen flow at 600 degrees C. Metals.

[11] 11Fan, L. *et al.* Corrosion behavior of Ti60 alloy under a solid NaCl deposit in wet oxygen flow at 600 degrees C. *Scientific Reports* **6**, 10.1038/srep29019 (2016). 10.1038/srep29019 PMC4928051 27357732

[12] Pettersson J, Asteman H, Svensson JE, Johansson LG (2005). KCl induced corrosion of a 304-type austenitic stainless steel at 600 °C; The role of potassium. Oxid. Met..

[13] Pettersson C, Pettersson J, Asteman H, Svensson JE, Johansson LG (2006). KCl-induced high temperature corrosion of the austenitic Fe–Cr–Ni alloys 304L and Sanicro 28 at 600 °C. Corros. Sci..

[14] Pettersson C, Johansson LG, Svensson JE (2008). The influence of small amounts of KCl(s) on the initial stages of the corrosion of alloy sanicro 28 at 600 °C. Oxid. Met..

[15] Jonsson T (2009). The influence of KCl on the corrosion of an austenitic stainless steel (304L) in oxidizing humid conditions at 600 °C: a microstructural study. Oxid. Met..

[16] Pettersson J, Svensson JE, Johansson LG (2009). KCl-Induced corrosion of a 304-type austenitic atainless ateel in O_2_ and in O_2_ + H_2_O environment: the influence of temperature. Oxid. Met..

[17] 17Jonsson, T., Folkeson, N., Svensson, J. E., Johansson, L. G. & Halvarsson, M. An ESEM in situ investigation of initial stages of the KCl induced high temperature corrosion of a Fe-2.25Cr-1Mo steel at 400 degrees C. *Corrosion Science* **53**, 2233–2246, 10.1016/j.corsci.2011.03.007 (2011).

[18] Shu Y, Wang F, Wu W (1999). Corrosion behavior of Ti60 alloy coated with a solid NaCl deposit in O_2_ plus water vapor at 500–700 ˚C. Oxid. Met..

[19] Dumas P, Stjohn C (1976). Nacl-induced accelerated oxidation of a titanium alloy. Oxid. Met..

[20] Yao Z, Marck M (1995). NaCl-induced hot corrosion of a titanium aluminide alloy. Mater. Sci. Eng., A.

[21] Johnson OW, Paek SH, Deford JW (1975). diffusion of h and d in TIO2 - suppression of internal fields by isotope-exchange. J. Appl. Phys..

[22] Fowler JD, Chandra D, Elleman TS, Payne AW, Verghese K (1977). T Diffusion in AL2O3 and BEO/D BEO. J. Am. Ceram. Soc..

[23] Douglass DL, Kofstad P, Rahmel A, Wood GC (1996). International workshop on high-temperature corrosion. Oxid. Met..

[24] Wang C, Jiang F, Wang F (2004). Corrosion inhibition of 304 stainless steel by nano-sized Ti/silicone coatings in an environment containing NaCl and Water Vapor at 400–600°C. Oxid. Met..

